# Remission from Depression in the DSM: Moving from Rhetoric to Restoration

**DOI:** 10.1007/s10615-017-0635-4

**Published:** 2017-06-28

**Authors:** Paige Gesicki, Holly Nelson-Becker

**Affiliations:** Social Work, Department of Clinical Sciences, Brunel University London, Kingston Lane, UXBRIDGE, UB8 3PH UK

**Keywords:** Language, Depression, Remission, DSM-5, Narrative theory, Mood memoirs, Metaphor

## Abstract

The Diagnostic and Statistical Manual of Mental Disorders, 5th Edition, the most recent edition of the Diagnostic and Statistical Manual of Mental Disorders, uses the term “remission” to describe the reduction of depressive symptoms. This paper argues that by categorizing someone who no longer has depressive symptoms as “in remission,” that person may feel indefinitely tied to his or her diagnosis. Considering the unfortunate stigma associated with mental illness, permanent linkage to diagnosis through records and professional memory may cause individuals to internalize pathology. In fact, the language of the diagnosis can affect self-perception in sensitive souls for a lifetime. As an implication for practice, we propose that cognitive and narrative therapy approaches, mood-memoirs, and use of metaphor present alternative uses of language that can reduce power imbalances between clinicians and clients, providing a bridge to healing.

Power is integrated in the use of language through the subtle ways in which words are chosen and operationalized (Crawford et al. 1999). However, words alone are not fully adequate to describe lived experience and the accompanying emotional sensibility and awareness. Despite this, language, a socially constructed system of symbols and codes, is the elemental way that humans attempt to communicate their experiences, including the pervasive mood state of major depressive disorder. This article identifies the impoverishment of the term depression, the lack of clarity in the use of the medical idiom “remission,” and introduces examples where cognitive and narrative theory can mitigate against the potentially harmful effects of diagnosis of major depressive disorder in remission. These approaches—the clinical practice of cognitive and narrative therapy, mood memoirs, and empowering metaphors—encourage an individual to be the protagonist of his or her own life story.

The importance of language as it pertains to mental health is made clear in examination of the Diagnostic and Statistical Manual of Mental Disorders, 5th Edition (DSM-5), the canonical text on mental health diagnoses. Mental health professionals refer to the dynamic list of criteria in the DSM-5 to diagnose individual psychopathologies and through diagnosis, to treat them. Diagnoses are undoubtedly helpful to clinical work because they provide a standardized description of the individual’s “problem” which thereafter guides clinical treatment (Ishibashi 2005; Kupfer 2005). If a mental health disorder can be categorized, then the assumption is that it can be managed. Upending the traditional power dynamic that favors mental health professionals over clients, this article emphasizes the perspective consistent with clinical social work that supports the wisdom of the diagnosed instead of the sovereignty of the diagnosor. Further, we examine how the language of the DSM-5 shapes an individual’s subjective experience of his or her mental health state, particularly depression. Although questions about clinical use of the term remission could apply to other clinical DSM-5 diagnoses, this paper will delimit discussion to address this concern through the lens of depression. This showcases specific applicable interventions for depressed mood that do not privilege the idea of possible recurrence nor obstruct a daily life from being lived in freedom from anxiety about a recurrence.

## Defining Depression and its Impact on Self-Perception

In the DSM-5, major depressive disorder “represents the classic condition in this group of [depressive] disorders,” unifying the other depressive disorders through the common characteristics of “the presence of sad, empty or irritable mood, accompanied by somatic and cognitive changes that significantly affect the individual’s capacity to function” (American Psychological Association 2013, p. 155). The diagnostic language of the DSM-5 attempts to describe the experience of depression in words that are accessible to mental health professionals through standardization of language; however, the words feel too far removed and flat to describe the reality of the psychological phenomenon (Gesicki 2015). Unlike disease states of the physical body, the presence of major depressive disorder lacks easily observable and definitive biological markers and is therefore difficult to consistently assess through physical symptoms. Diagnosis in mental health thus remains an art as well as a science. Currently, the means of detecting depression are limited to subtle molecular and neural changes that are complicated to read and not yet unanimously endorsed (Krishnan and Nestler 2010).The path of major depressive disorder is often tracked through oral and written accounts—the development of the illness is relegated to the malleable world of words. Language serves as a signifier of experience, but is not always fully capable of explaining that experience.

The linguistic shortcomings to describe depression are not limited to the DSM-5. Levitt et al. (2000) studied the use of “burden” metaphors used by clients in therapy as they attempted to describe their experience of depression. The study acknowledges that the subjective experience was so difficult to verbalize that clients turned to descriptive metaphors in their attempt to “more accurately capture the quality of an emotion [instead of using] an adjective or an emotional label” (Levitt et al. 2000, p. 24). Our clients have described depression with strong perceptual images, such as: “a fog so dark I cannot see through it,” or a “glass wall between me and the rest of the world,” often using rhetoric of physical pain such as “You know when your circulation gets cut off in your leg from sleeping wrong? That’s how my emotions feel; I’m numb to the touch and inside the pain is excruciating.”

The word “depression” has become so diluted in mainstream English vernacular that it not only grossly fails to describe the lived experience of a clinical depression, but it also has assumed a distinctly different meaning in its common use. Pulitzer Prize winning author William Styron describes the “semantic damage” of the misuse of the word, which “has slithered innocuously through the language like a slug, leaving little trace of its intrinsic malevolence and preventing…a general awareness of the horrible intensity of the disease” (Styron 2007, p. 37). In his memoir, *Visible Darkness*, Styron (2007) describes his frustration with the descriptive limits of language, as well as a brief demonstration of the evolution of words in their social construction. “When I was first aware that I had been laid low by the disease major depressive [disorder], I felt a need, among other things, to register a strong protest against the word ‘depression’” (Styron 2007, p. 136). Styron suggests that the much older term melancholia better represents the darkness of the disease. Melancholia is a term which dates to the time of Hippocrates and has origins in the black bile, one of the four humors (Nelson-Becker 2017). Styron submits that the term depression is tagged with a “blank tonality” that “lacks magisterial presence.” Further, he alludes to other related meanings such as “economic decline or a rut in the ground,” calling the word “a true wimp” for the hugeness of what depression means.

### Problematic Terms: Remission and Recovery

Remission is a common term used in many DSM diagnoses. For clarity, this term is explored here in relationship to depression rather than other mental illnesses where it also may apply. A salient question is whether the term “remission,” defined as symptom resolution, should be included in such a prescriptive text as the DSM-5 which, because of its widespread medical authority, has the power to affect a diagnosed individual’s sense of self (Gesicki 2015). Qualifying someone’s recovery as “in remission,” the clinician robs that person of his or her ability to be fully healed; the experience of depression may no longer be viewed as a transient stage, but instead a fixed state of being or a pathological life sentence.

The concept of recovery also connects clients to the question, recovery from what? Recovery, used by some mental health agencies, such as the National Alliance on Mental Illness which hosts “recovery groups,” can be problematic as this language suggests a constant state about which someone must remain vigilant rather than a period of time with an endpoint in successful management. While illness will always be a part of an individual life story, how it is integrated into a sense of self in the present and the meaning it denotes carry implications for future health status.

The term remission is a poor measure to describe a person’s relationship to major depressive disorder for numerous reasons; at least, it is logically inadequate, and at worst, it is intimately damaging. First, measuring recovery on the basis of a decrease of negative symptoms on a standardized list is problematic because major depressive disorder cannot be measured objectively (Zimmerman et al. 2006). The Hamilton rating scale, like many other common tools used to define depression such as the Geriatric Depression Scale, is an ordinal scale, and distance between response items is arbitrary. Thus, the outcome of intensity regarding depressed mood is subjective overall.

Secondly, “remission” is an idea constructed by medical and mental health teams, yet studies show that people who have actually experienced depression believe that remission does not accurately reflect the lived experience of recovery, which is less about the absence of challenging symptoms and more about the presence of positive qualities (Zimmerman et al. 2006). Some studies provide evidence that initial response to pharmacological and nonpharmacological treatment predicted ultimate recovery rates as well as who remained well (Kupfer 2005). Finally, and most importantly, since language has the power to affect one’s thought and behaviors, diagnosing someone as “in partial or full remission” risks the possibility of that individual feeling permanently tied to his or her pathological diagnosis. This descriptor has the consequence of reminding the individual, as well as their loved ones, of his/her former non-normative psychological state even after full recovery.

Once diagnosed, records documenting depression will generally follow a client to therapy. They are also often present on each hospital readmission whether that readmission is related to a mental health or medical concern. Even if a client has learned skills to keep depression at bay, the specter of re-emergence can feel oppressive. The client may well wonder whether he/she can ever move beyond professional memory. Therapists may argue this provides safety for the client, but clients often sense this as constriction. Thus, clients are at times unfairly stigmatized based on psychiatric or health records indicating a depression diagnosis that follows them into the future.

Mary Jane had an experience of Major Depressive Disorder (MDD) when she was in her early 30s. In her mid-40s, she was hospitalized for knee surgery after taking up running in part to keep depression at bay which she had successfully accomplished. However, on scanning her documentation, her surgeon prescribed her an anti-depressant medication without asking her. This was a medication she did not want; the episode caused her to feel marginalized and not included in her own care.

If remission is the primary objective of clinical treatment, the question should be posed: What exactly characterizes remission in the realm of mental health and specifically depression? In 1988, the MacArthur Foundation Research Network on the Psychobiology of Depression organized a conference to review and tighten the definition of many “recovery” terms, including the term remission (Frank et al. 1991). After acknowledging the considerable inconsistencies across views of the course of depression, the task force agreed that remission would thereafter refer to an individual who is asymptomatic for a brief duration, which can occur spontaneously with or without treatment (Moller et al. 2011). Symptomatic does not mean the complete absence of symptoms. Instead, it is defined as the presence of no more than minimal symptoms, as proven by a score of 7 or lower on the 17-item Hamilton Depression Rating scale (Zimmerman et al. 2006).

Judging a person’s progress on any ordinal scale such as the Hamilton depression rating scale is problematic because the measure has been criticized for numerous reasons, including its lack of empirically derived cutoff points (Ballesteros et al. 2007). There are no “empirically driven” biological markers to prove the presence or absence of depression. Instead, mental health is determined by a continuum that begins in exceptionally high (or low depending on the measure) group scores and individual complaint, and ends in reference to general population norms regarding mental and emotional health or flourishing. The DSM-5 itself concedes that, “although an extensive literature exists describing neuroanatomical, neuroendocrinological, and neurophysiological correlates of major depressive disorder, no laboratory test has yielded results of sufficient sensitivity and specificity to be used as a diagnostic tool for the disorder” (American Psychological Association 2013, p. 165).

Across mental health and medical fields, if a patient is deemed “in remission,” the individual is not necessarily free of the illness; instead, the phrase implies that the illness has abated temporarily and may return. Therefore, if someone is diagnosed with depression in full remission, the implication is that they have not experienced any “significant signs or symptoms of the disturbance” in the past 2 months (American Psychological Association 2013, p. 188). The DSM-5 relies even further on the measurability of major depressive disorder by providing the additional qualifier of “partial remission,” which is when symptoms are present but full criteria are not met for major depressive disorder (American Psychological Association 2013, p. 188). Likewise, the grassroots mental health advocacy group NAMI, the National Alliance on Mental Illness, defines depression as “a life-long condition in which periods of wellness alternate with recurrences of illness” (Duckworth and Shelton 2012, p. 1). Thus, it follows that major depressive disorder, according to the DSM-5 and NAMI, a preeminent advocacy group, is not a transient mood state from which a person can fully recover. Yet people do and have recovered.

Although it is true that individuals with major depressive disorder often experience recurring depressive episodes, adding the qualifier “in remission” to the diagnosis, instead of eliminating the diagnosis entirely, can be perceived as pathologizing (Duckworth and Shelton 2012). The individual is now medically regarded as a “depressive in remission” instead of an individual who once suffered or occasionally suffers from a depressive episode. “In remission” suggests that the illness will return. It defines a person by a once-held illness. This makes it hard to integrate depression as a past episode of one’s life story, due to the looming fear that an episode will reappear at any given time. Clients may remain over vigilant. Instead of being rid of the pathology altogether, the label remains with a qualifier, which can inform the way future health professionals guide conversations with the diagnosed individual. This is not a strengths-based conceptualization. However, the process of constructing a new self-narrative is highly individualized and it is possible that some who struggle with depressed mood are less sensitive to linguistic cues. For these individuals, incorporating the idea *in remission* in a positive way consistent with the dominant professional community discourse is possible.

## Implication for Practice: Cognitive Therapy, Narrative Therapy, Mood Memoirs, and Empowering Metaphors

The DSM-5’s medical model aligns with modernist approaches, which prioritize objectivity and tend to be more diagnostic in nature, often sustaining a power dynamic with therapist as expert and client as a subject (Ishibashi 2005; Nelson-Becker et al. 2013); therefore, when a clinician applies a particular diagnosis to an individual, he or she utilizes a rhetorical currency that only privileged professionals can speak with authority, excluding others from the conversation. The resulting power dynamic directly contradicts the National Association of Social Workers Code of Ethics, which emphasizes the importance of egalitarian partnership between professional and nonprofessional as critical for change (NASW Code of Ethics 2008). Cognitive therapy, narrative therapy, mood memoir writing, and engagement with metaphor are methods for changing the paradigm of depression from one of victimization to one of vanquishment.

Considering the prescriptive quality of language when issuing a diagnosis, it is important to look critically at the language used in the DSM-5 and helpful to view this diagnostic manual through the lens of cognitive and narrative theory. Research affirms the value of both cognitive and narrative approaches in clinical work with depressed clients over control groups (Lopes et al. 2014; Vromans and Schweitzer 2011). Cognitive and narrative therapy both offer linguistic strategies for identifying and changing self-talk and stories about the self. Cognitive work is often used to change immediate responses, thereby changing present and future behavior, while narrative work at times takes a wider scope, looking at narratives from the past and present. It is possible that narrative therapy may require more cognitive resources to enter into meaning making compared to cognitive therapy that offers assistance through application of specific techniques. However, cognitive and narrative therapy both may be applied in ways that equalize the power dynamic, consistent with a clinical social work approach that upholds treatment as partnership.

### Cognitive Therapy

Cognitive therapy produces outcomes that point to the danger of applying an unshakeable diagnosis. For example, according to cognitive theory, someone with depression is operating from dysfunctional schemas they have created. Distorted schemas are the foundations of a person’s mindset from which all thoughts follow, or the way they make meaning of their world; therefore, if the schema is self-critical and negative, the thoughts are tailored to be “schema-congruent” and are similarly harsh. The schemas initiate and reinforce negative views in an insidious vicious cycle of distorted information processing and furthermore, these have been shown to prompt and sustain depression (Beckerman and Corbett 2009). Pessimistic thoughts are assumed to be the reality, but instead often represent the exaggeration of reality. Viewed through cognitive theory, the intrusive thought that one is diagnosed with depression, or even depression in remission, can reinforce deeply held negative beliefs, furthering these pessimistic roots through the sheer repetition of loaded words (Beckerman and Corbett 2009).

One example of a client who struggled with intrusive pessimistic thoughts was Amelia, a 35-year-old engineer. Amelia had struggled with depression for many years. Her primary intrusive automatic thought was that she could never do anything right and would always fail no matter what she tried. This caused her to hold back from making suggestions at work that could have significantly helped her career. Instead, suggestions from others were often implemented, even when Amelia clearly conceptualized potential problems. Her therapist, using cognitive therapy, taught her to explore the evidence for her belief and substitute the idea that offering her suggestions could be in itself a reward. Gradually, she began to see herself as capable of sharing on an equal level with colleagues and having the courage to try alternative behaviors. She moved beyond self-defeating cognitions and began to view herself as separate from her depression. Gradually she advanced in her company as her colleagues increasingly relied on her advice. Although she occasionally still suffered from a depressed mood, she was able to balance this with evidence of her value, substituting words in her self-talk that provided a more realistic self-appraisal.

### Narrative Approaches to Treatment of Depression

A narrative approach contends that an individual’s problem is socially constructed through language, a formal system of symbols and codes, and therefore the problem should be resolved through language (Coady and Lehmann 2008). When applying narrative theory to the experience of clinical depression, the question arises: Is it possible to generate a new meaning for the words used in the clinical description of depression, a meaning that is restorative and healing instead of incarcerating and heavy? Narrative therapy is a clinical model based on narrative theory, which purports that through supportive dialogue, judgment-laden, problem-saturated stories can be replaced with stories of strength, accomplishment, and courage. The self-critical story can be rewritten to one of hope (Coady and Lehmann 2008). This type of therapy aligns with narrative theory’s assertion that language is not an individual endeavor—problems are created through discourse with others and therefore should be addressed in conversation with others.

Diagnosis is about internalizing problems and identifies the responsibility that individuals assume for their participation in treatment. In narrative theory, a client who self-identifies as “in remission” per a clinician’s externally-imposed diagnosis is providing fuel to the problem, therefore keeping the depressive identity alive. Narrative therapy suggests that when problems are externalized, people can see themselves as separate from their illness and then can better work towards *restoration* of self. This created self is a beautiful mosaic that lies beyond remission and recovery to occupy a new space of greater self-knowledge. Since the process of constructing a new story of one’s life is a highly nuanced, individualized process, a story of oneself which includes being “in remission” from depression may be experienced as empowering and even incorporated positively into one’s new narrative.

With a narrative approach, values and hopes are traced to specific moments and events. Once these are discovered or re-discovered, the attributes which more closely define an individual and who they choose to be can be included in conversations to assist clients to “re-author” or “re-story” their experience. For a narrative theorist, this would entail deconstructing the client’s reality and creating a new conversation. The truth of the story (the fact of past depression) is less important than an ability to revise interpretations in order to better cope in the present and future (Ridge 2009). Consistent with the theory, it is important to recreate an empowering alternate story through conversation or discourse, either spoken or written, because language gains power in its exchange with another. For example, leading narrative clinician David Epston wrote a summarizing letter to his clients after most interviews, which served as a clinical note. In doing so, Epston created a shared body of knowledge that dispersed power between professional and client and promoted an egalitarian relationship (White and Epston 1990). This was a model for therapeutic engagement and treatment, facilitating client independence and growth. Skillful sharing of stories can heal.

This work then becomes an act of resistance to problems, or at least what the client has defined as problematic. Narrative approaches are a good antidote to the aspect of major depression in which an individual interprets and experiences the world through inflexible negative frameworks. One example of a narrative approach used specifically to treat major depression is the use of innovative or sparkling moments that emerge in therapeutic dialogue in which the client offers exceptions to his/her problems. Clinicians who track client transformation through narrative markers guide psychotherapy by reminding the client of innovative moments (exceptions to the problem) until these moments are repeated frequently enough to replace a maladaptive framework with a functional one (GonCalves et al. 2015).

An example of narrative work is illustrated by Jacob and his clinician. Jacob was a 27-year-old middle school science teacher who viewed himself as particularly inept in social relationships with peers, especially women. Growing up, he had often internalized negative messages about his social skills given by his mother. As a result, whenever someone showed interest in him, be became flustered and began to stutter. His clinician asked him to recall a time when he experienced something different. After some thought, he realized that when he was talking with someone about the physics of black holes and dark energy, he spoke fluidly and kept his listener spell-bound. They explored this image further—an innovative moment—and Jacob saw that his focus on something outside himself combined with his knowledge allowed self-confidence to emerge. He also began to consider specific times in his youth when he lacked self-confidence. Often, these had been times when his mother had hovered nearby, expecting him to struggle. He began to identify and name her actions as an external source of his learned behavior and thus capable of transformation. He could alter his perception of her as an expert in relational matters and locate others who could serve as models. When he recognized his conversational skill with women as something he could change rather than an immutable quality, he constructed a new self-description with words that served to inspire him to move into that new self.

Narrative work looks for unique outcomes in the relationship of the problem to the person. A key task is to open space for new ways of being, thinking, and performing. This is often done through re-membering events to highlight alternative ways of constructing their unfolding and to underline client strengths, which are usually minimized in the face of a diagnosis of depression, particularly one where the ready solution is pharmacological.

While depression may have biological foundations, neuroscience is discovering more about how new synapses are created through ways people act or think, suggesting that people can clearly have an effect on their own illness. Through their support of research on complementary and alternative medicine, the National Institutes of Mental Health (NIMH) and Health (NIH) propose there is more to learn about body-mind connections on the path to full health of all kinds (Shinohara et al. 2013).

### Mood Memoirs

The dialogical imperative of narrative work introduces the third method of healing—the mood memoir. A private journal can be cathartic to write, but explaining an experience in one’s own words and sharing that with a willing listener or reader is powerful because of its discourse. Memoirs of depression, such as Joan Didion’s *The Year of Magical Thinking*, Kay Redfield Jamison’s *An Unquiet Mind* and the aforementioned William Styron’s *Visible Darkness* confront mental illness stereotypes and stigmas by providing an alternative, truer story (Kramer 2005). Through mood memoirs, individuals are invited to externalize depression as a problem to be faced and overcome, instead of an innate character flaw or unavoidable life sentence.

Rhetoric scholar Pryal (2010) describes the “mood memoir” as a literary genre that provides a space for those with mood disorders, who may be otherwise deemed as illegitimate rhetorical sources, to gain power through telling their stories. She argues that “mood memoirs can be read as narrative-based responses to rhetorical exclusion by the psychiatrically disabled” (p. 480). Pryal’s work outlines a new genre; however her point about gaining authority through the written word is salient to the idea of empowerment through creating one’s own story. Many clinicians outside of the narrative school of thought, such as cognitive behavioral therapists, encourage clients to use journaling as a supplement to treatment in order to clarify and gain ownership over their experiences (Smith et al. 2000). While not everyone is drawn to write, for those who are, this can be a wonderful means of self-discovery and healing. The possibility of new understanding is probably why the everyday use of diaries has been a compelling means of detailing events and feelings throughout recorded time.

Styron’s memoir is characterized as a mood memoir for its in-depth description of his experience of depression, which he calls “the disease.” Styron demonstrates the healing benefits of externalizing the problem, a strategy that is often utilized in narrative therapy. He successfully used his mastery of language to gain enough distance from his debilitating experience of the mood disorder to write an illuminating first-hand account. According to the DSM-5, Styron might have been characterized as *in remission* from his major depressive disorder, which would be a treatment success, since experts “suggest that achieving remission of symptoms should be viewed as the primary goal [of treatment]” (Zimmerman et al. 2012, p. 78). However, in light of his criticism about the limitations of language to adequately describe the complexity of depression, Styron himself would likely not describe his own journey through depression in the static, pithy terms of “remission.” Furthermore, the use of writing as an avenue of healing, by Styron and diary-keepers of more mundane nature, illustrates the clinical application of Pryal’s (2010) argument that power can to be restored to the individual in treatment through rhetoric.

### Empowering Metaphors

A fourth possibility besides cognitive therapy, narrative therapy and mood memoirs is the use of empowering metaphors for clients with depression to use in self-description. Metaphors have the power to engage the minds of clients as well as their spirits. While depression robs clients of inspiration and hope for change, metaphors open avenues back to illumination of all that is still possible. Metaphors, through the language of exception, can re-orient and distill attention back to what is essential for a client. They give voice to what was diminished and depleted. When voiced or named, the challenge is no longer so large that it can’t be addressed and altered. Metaphors thus build resilience for people who have lost track of themselves and their hopes for who they thought they could be.

One example of a powerful metaphor is the idea of the client as hero or heroine (Campbell 1968; Duncan et al. 2013). The hero’s journey was detailed in work by Campbell (1968) who sought to synthesize major myths across cultures. Through working with the hero metaphor, clients are re-positioned in their rightful place as owner and director of their lives. How would this metaphor work in practice? The client is ensconced in an *Ordinary World*, which has become a world of suffering for him or her. The hero is discussed against the background of person (individual characteristics) and environment (constraining or facilitating variables).

The therapist assists the client to hear the *Call to Adventure*, which is the idea, or even the certainty, that suffering can decrease and life can change. The *Call to Adventure* is fully acknowledged when something shakes up the status quo (outside factor) or the appeal emerges from within (inside factor). The depressed person will often first *Refuse the Call* out of fear and turn away. However, with the help of a social worker, counselor, psychologist, or other mentor, the client gains the courage to *Cross the Threshold* into unfamiliar terrain. There are always *Tests, Allies, and Enemies* in this path. The particular faces of these features can be identified by the depressed person with assistance from his/her mentor. The client releases his/her former self (the self with depression), which represents facing the *Ordeal*. The *Reward* might be said to be newly-found resilience and restored mental health through psychotherapeutic or social work. The *Road Back* would involve re-integration with ordinary life but with expanded knowledge, self-understanding, and competence. The *Elixir* consists of new patterns and habits that, having transformed the client, can also be used to assist others with the new knowledge gained from the struggle. The *Elixir* also mitigates against defeat from new tests and serves to strengthen and sustain the hero or heroine with depressed mood his or her whole life long.

Clients who suffer from depression may unnecessarily minimize or distort images of themselves as strong and capable. Geoffrey, a 50-year-old businessman, worked with his therapist to counter his depressed mood by creating a preferred view of himself as hero of his own story. The idea that he could be a hero to whom others might look as a role model helped give new perspective to his life. He was raised in an abusive family where any sense of self-worth he held was devalued and then destroyed. His approach had been to largely disappear in public arenas, particularly when it would have furthered his career to speak publicly about work. The metaphor of the hero’s journey helped Geoffrey form a new identity and resist depressive thoughts. As he changed, his colleagues also began to change their interactions with him.

## Conclusion

The term remission in the diagnostic criteria for major depressive disorder may be problematic for numerous reasons, including its attempt to apply an empirical standard to an idiosyncratic mood disorder, as well as the potentially destructive implication that an individual can never fully recover from depression. The term *in remission* for some may hold open space to heal and be incorporated positively into a new narrative. For others, it suggests that one cannot move beyond the illness. Cognitive and narrative theory aid in the examination of the DSM-5 by providing a lens through which to view the linguistic repercussions of the text for individuals diagnosed with major depressive disorder.

Narrative theory contends that language provides a system for us to make meaning of our experiences. When clients are told by a mental health professional with a privileged vocabulary they cannot recover from a painful disorder, but instead should expect to live in a limbo vacillating between full and partial remission, that story may be internalized to become one’s fate. In order to counteract this loss of agency at the hands of misused language in the DSM-5, cognitive therapy, narrative therapy, mood memoirs, and metaphors provide empowering autobiographical counter narratives to the limiting stories about mental illness offered by psychiatrists, some therapists, policymakers, and the general public.

Perhaps an additional problem lies in who has the “last word” in establishing recovery or a lack thereof. In his article asking how depression should be defined in the depressed patient’s perspective, Zimmerman et al. (2006) strays from the traditional “medical model” in favor of a client-centered perspective, which is well-aligned with the social work paradigm. He found that patients who deemed themselves free of the depressive state reported that positive markers of mental wellness, including “optimism, vigor, and self-confidence” were more accurate indicators of remission than the absence of symptoms (Zimmerman 2006, p. 150). Therefore, it is not what once was that is now missing, it is what has been found and added that matters. Further, experts on depression, individuals who experienced it themselves, agreed that the DSM-5’s definition does not accurately reflect lived experience. Even arguably positive terms such as “remission” versus “diagnosed” can be limiting due to the inequality of power inherent in the act of naming an experience that is not your own. People with depression, regardless of specific clinical diagnosis, who work with it successfully do more than recover themselves to a state of pre-diagnosis—they stand in a new and different place as a new version of themselves. Their story is not merely resuscitation of a former self, but instead one of resurrection into a new self.
